# Molecular signatures of organic particulates as tracers of emission sources

**DOI:** 10.1007/s11356-022-21531-0

**Published:** 2022-07-25

**Authors:** Angelo Cecinato, Alessandro Bacaloni, Paola Romagnoli, Mattia Perilli, Catia Balducci

**Affiliations:** 1 National Research Council of Italy, Institute of Atmospheric Pollution Research (CNR-IIA), 00015 Monterotondo, RM Italy; 2 Dept. of Chemistry, University Roma-1 “Sapienza”, Rome, Italy

**Keywords:** Molecular signature of sources, Diagnostic concentration ratios, Particulate organic matter (POM), Air pollution, Toxicants

## Abstract

Chemical signature of airborne particulates and deposition dusts is subject of study since decades. Usually, three complementary composition markers are investigated, namely, (i) specific organic compounds; (ii) concentration ratios between congeners, and (iii) percent distributions of homologs. Due to its intrinsic limits (e.g., variability depending on decomposition and gas/particle equilibrium), the identification of pollution sources based on molecular signatures results overall restricted to qualitative purposes. Nevertheless, chemical fingerprints allow drawing preliminary information, suitable for successfully approaching multivariate analysis and valuing the relative importance of sources. Here, the state-of-the-art is presented about the molecular fingerprints of non-polar aliphatic, polyaromatic (PAHs, nitro-PAHs), and polar (fatty acids, organic halides, polysaccharides) compounds in emissions. Special concern was addressed to alkenes and alkanes with carbon numbers ranging from 12 to 23 and ≥ 24, which displayed distinct relative abundances in petrol-derived spills and exhausts, emissions from microorganisms, high vegetation, and sediments. Long-chain alkanes associated with tobacco smoke were characterized by a peculiar iso/anteiso/normal homolog fingerprint and by *n*-hentriacontane percentages higher than elsewhere. Several concentration ratios of PAHs were identified as diagnostic of the type of emission, and the sources of uncertainty were elucidated. Despite extensive investigations conducted so far, the origin of uncommon molecular fingerprints, e.g., alkane/alkene relationships in deposition dusts and airborne particles, remains quite unclear. Polar organics resulted scarcely investigated for pollution apportioning purposes, though they looked as indicative of the nature of sources. Finally, the role of humans and living organisms as actual emitters of chemicals seems to need concern in the future.

## Introduction

Chemicals released into the atmosphere are known as posing a threat for humans and injuring the environment. Therefore, the knowledge of nature, amount, and land spread of emissions is mandatory whenever legislative or technological actions must be implemented to mitigate the toxicants’ impact (Albaiges et al. 1984; Bascom et al. 1996; Yassaa et al. 2001; Ma and Harrad 2015; Błaszczyk et al. 2017; Sifakis et al. 2017; Cetin et al. 2018; Nieder et al. 2018; Vaz 2018). Chemical and physical characterization of both gaseous and particulate phases plays a key role to picture the behavior of pollutants in the environment (i.e., through valuing the concentrations of selected chemicals in air and exhausts, and comparing them with current legislation). Besides, dedicated studies allow assessing the relative importance of pollution sources that affect the sites or land domains subject of study (Gundel et al. 1993, Hecht 1999; Ventrice et al. 2013, Jedynska et al. 2014a,b, Giulivo et al. 2016, Liu et al. 2017, Praveena et al. 2018, Rabhi et al. 2018, Yury et al. 2018, Brehmer et al. 2020).

The first approaches to identification of the emission sources of organic toxicants by means of molecular signatures were carried out in late twentieth century (Daisey et al. 1986; Harrison et al. 1996). Attention was paid overall to alkanes, PAHs, and nitrated derivatives (NPAHs); however, other groups were taken in account also, including fatty acids, halides (polychlorinated dioxins/furans [PCDD/Fs], polychlorobiphenyls [PCBs], and polybromodiphenyl ethers [PBDEs]), polysaccharides (levoglucosan, mannosan), sterols (cholesterol, stigmasterol, sitosterol), and triterpenols (amyrins). According to these studies, the characterization of organic substances comprised in particulate matters (both airborne particles and dust settled on surfaces and soil) resulted an important tool of investigation but also a challenge for scientists, due to complex nature of these matrices. In fact, organic compounds display a wide variety of chemical and physical properties, e.g., acidity/alkalinity/neutrality, rate of polarity, solubility in water and n-octanol, vapor pressure, resistance to action of oxidants, and light. As for chemical composition, organics include linear and cyclic aliphatic hydrocarbons, polycyclic aromatic hydrocarbons, fatty acids and phenols, amines, carbonyls, organic halides, sulfates, and phosphates.

Many organic compounds have been associated to distinct emission sources and recognized as tracers of living organisms, natural phenomena, and man’s activities. Unfortunately, only in few cases one substance is unequivocally typical of one only emission and allows assessing the impact of that source onto the environment. Usually, chemicals occur in more types of emissions, so that the peculiarity is lost; nevertheless, in this case the composition per groups and the distribution pattern of congeners within each group can aid in identifying the pollution sources. For instance, biofuels are usually richer of esters than fossil fuels, and the reverse occurs with regard to polycyclic aromatic hydrocarbons (PAHs) (Damanik et al. 2018). Besides, the percentages of PAHs associated to ultra-fine, fine, and coarse fractions of suspended particulate are a function of the nature of source (Zielinska et al. 2004). Fresh exhausts undergo the action of light (overall UV) and oxidizing species (O_3_, NO_2_, OH, and NO_3_ radicals) (Arey and Atkinson 2003; Estève et al. 2004; Perraudin et al. 2007; Chu et al. 2010). Therefore, chemicals released primarily into the atmosphere tend to change into degradation products; e.g., alkenes and PAHs into ketones and quinones, polyacids, diols, epoxides, cumulatively defined as secondary pollutants. These transformations alter both chemical signature of the substrate and toxicity of the air parcel impacted by the emissions (Atkinson and Arey 1994; Durant et al. 1999; Bandowe et al. 2014). Reactivity of compounds introduces some rate of uncertainty with regard to identification of emission sources of airborne particulate. On the other hand, due to wide ranges of lifetime characterizing organics, the rank of decomposition can work as an index of aging, i.e., of the importance of processes developing in the atmosphere (Sofowote et al. 2010; Cecinato et al. 2014). With regard to deposition dust, reactivity seems to play a twin role. On the one hand, its high surface extension favors the adsorption of chemicals from the air; the substances are back released when the contour conditions are favorable, the substrate composition changes, and this latter acts as secondary emitter. On the other hand, the collection time of depositions adopted for chemical characterizations is ≥ 15 days, which implies the wide occurrence of decomposition products of primary pollutants.

According to the above considerations, the simple approach of molecular signature of environmental particles suffers some intrinsic constraints; hence, better tools are employed today to trace the emissions, e.g., principal component analysis. Nevertheless, the knowledge of chemical fingerprints remains suitable as a preliminary screening of factors forcing pollution; for instance, chemical profiles of POM (and the resulting numerical parameters) allow excluding or including as real main pollution sources the types of emissions suspected to affect the environment. Molecular signature is easily integrated in statistical approaches based on a number of chemical and physical variables (Kavouras et al. 2001, Mostert et al. 2010, Brown and Brown 2012a, 2012b, Khedidji et al. 2017, Chen et al. 2019, Maechler et al. 2019, Molnar 2019, Sofowote et al. 2020), which look fine for source apportionment studies. Besides, any multivariate analysis approach alone does not add any contribution to knowledge of the nature of sources, whereas no preliminary information is available about the chemical fingerprint of emissions. For instance, multivariate analysis is able to gather or distinguish sets of samples and of chemical species within environmental databases, by putting in the evidence differences and similarities among them. Nevertheless, it is necessary knowing the chemical profiles of emissions and other contour information (e.g., reactivity of compounds, type of locations, size, and chemistry of substrates…) to assign reliably the abovementioned behaviors to specific origins.

With regard to chemicals hosted, interiors as a whole are a space different at all from outdoor environment (Zhao et al. 2007, Guo and Kannan 2013, Sangiorgi et al. 2013, Romagnoli et al. 2014, Hassanvand et al. 2015, Tran et al. 2015, Oliveira et al. 2016, Subedi et al. 2017, Liu et al. 2018, Lu et al. 2018, Lucattini et al. 2018, Steinemann 2018, Wong et al. 2019, Zhu et al. 2019). Three categories of contaminants affect indoor locations, i.e., (i) chemicals released overall outside and driven indoors through building openings and ventilation devices (e.g., hydrocarbons comprised in motor vehicle exhausts); (ii) substances released indoors and outdoors at broadly analogous levels (e.g., nitrogen oxides, psychotropic substances); and (iii) compounds released typically indoors (e.g., cosmetics, plasticizers). Indoors, the substances released meet up reaction chambers with temperatures roughly steady along the whole year, ozone normally much less than outside, and surfaces much larger. These factors deeply influence the chemistry of locations, the lifetime of substances, and the gas/condensed phase equilibria. In particular, in interiors the substance ability to interact with the human body is different with regard to intensity and route, compared with open air. At this regard, it is worth noting that current legislation aimed at preserving health considers only inhalation as primary way of intake; meanwhile, outdoor pollution is viewed as predominant, and the occurrence of toxicants in interiors is linked to intrusion from outside. This is the reason why attention is paid usually to gasses and fine aerosols, as well as to [lung] cancer. Instead, indoors the neat exposure to toxicants is larger, and the amounts of dusts with which humans enter in contact exceed of orders of magnitude those of fine particles inhaled. Therefore, the alternative ways of body intake (i.e., skin contact and ingestion) gain importance (Xing et al. 2011; Hou et al. 2018; Weiss et al. 2018; Settimo et al. 2020a), and the role played by depositions increases, as well as that of health problems other than tumors. That garbles the role of toxicants affecting interiors and promotes the search for their sources.

This paper aims at providing a short review of current knowledge concerning the molecular fingerprints of particulates (both airborne and settled), suitable to elicit information about the sources of pollution. Three major categories of fingerprints are discussed, namely, (i) individual tracers; (ii) diagnostic concentration ratios; and (iii) homolog percent distributions within groups. As for chemicals, non-polar hydrocarbons (i.e., chain- and cyclo-aliphatic compounds) are examined in particular here, and glance is given to need of further investigations aimed at understanding the sources of uncommon alkane/alkene percent distributions. Finally, some insights are provided about the role played by living organisms and humans, as actual emitters of contaminants, with regard to chemistry of their own life places.

## The state-of-the-art of research about molecular signatures of pollution sources

### General features of emission profiles

Three key factors influence the composition of both anthropogenic and natural emissions, as it results from chemical analysis. They are (i) the operating conditions of source, including the kind of fuel, temperature of exhausts, and the type of abatement devices adopted; (ii) the collection procedure of exhaust (which includes vapors, condensation waters, and particle matters); and (iii) the methodology adopted to process samples and determine chemical composition. These factors hinder to assign thorough emission factors to chemicals released by sources, and precise chemical profiles to groups of substances like alkanes and PAHs (Tobiszewski and Namiesnik 2012; Cecinato et al. 2014). Investigations undertaken with different methodological approaches can lead to results hardly comparable (Kavouras et al. 1999). For instance, the profile of particulate n-alkanes actually identified in emissions depends on the effluent temperature during sampling operation, which influences the loss rates of the most volatile compounds. Analogously, the profile of airborne 3/4-ring PAHs depends on year season as well as on the use of the only filter membrane or also vapor trap to collect samples. Hence, the study of their percent distribution in the emissions and in airborne particulates is preferably restricted to high molecular weight homologs, namely, to hydrocarbons with carbon number ≥ 25 (C_25_). As for PAHs, compounds with vapor pressures of the same order of magnitude (e.g., fluoranthene/pyrene) or with high molecular weights (e.g., benzo[a]pyrene/benzo[ghi]perylene) are kept in consideration when exploring concentration ratios hypothetically diagnostic for source assessment purposes.

### n-Alkanes

Non-polar fraction of particulate organic matters (POM) includes numerous groups. They are alkanes, alkylated mono-aromatics and biphenyls, alkenes, branched and cyclic aliphatic hydrocarbons. Among them, attention has been paid overall to n-alkanes (linear homologs); alicyclic compounds have been investigated as tracers of petrol products, and mono-methyl substituted alkanes as markers of tobacco smoking. As total, n-alkanes are among the most abundant components of particulate organic matter. For a long time, investigations dealing with this group were restricted to chemistry of high plants (Eglinton et al. 1962; Eglinton and Atkinson 1967; Li et al. 2018) and to characterization of vehicle exhausts. Instead, n-alkanes gained concern when the strong dependence of their molecular imprinting on nature of source was ascertained, as well as their toxicity that includes skin inflammation, pulmonary edema, respiratory disfunction, co-carcinogenic and co-tumorogenic properties (Rabovsky and Judy 1989).

The saw-tooth distribution of high-molecular-weight n-alkanes has been associated to terrestrial high plants (Simoneit and Mazurek 1982; Alves et al. 2001; Rabhi et al. 2018). Indeed, biogenic synthesis leads to generation preferably of even C-numbered fatty acids; afterwards, acids tend to loose CO_2_ through the natural process of decarboxylation, and form odd-C numbered n-alkanes as the final products (or alkenes, in the case of unsaturated acid precursors). Since in the case of high trees this phenomenon is more evident along the range of long-chain homologs, the most used parameter to value the impact of (high) vegetation is carbon preference index starting from normal pentacosane (CPI_25_) (Alves et al. 2001; Pio et al. 2001; Omar et al. 2007). This parameter is expressed by the following formula (1):

1 $${\text{CPI}}{25}=\frac{\mathrm{ n}{\text{C}}{25}+{\text{nC}}{27}+{\text{nC}}{29}+{\text{nC}}{31}+{\text{nC}}{33}+{\text{nC}}{35}}{2*(\mathrm{n}{\text{C}}{24}+{\text{nC}}{26}+{\text{nC}}{28}+{\text{nC}}{30}+{\text{nC}}{32}+{\text{nC}}{34})}+\frac{{\text{nC}}{25}+{\text{nC}}{27}+{\text{nC}}{29}+{\text{nC}}{31}+{\text{nC}}{33}+{\text{nC}}{35}}{2*(\mathrm{n}{\text{C}}{26}+{\text{nC}}{28}+{\text{nC}}{30}+{\text{nC}}{32}+{\text{nC}}{34}+{\text{nC}}{36})}$$

where nC_*i*_ means the concentration of n-alkane homolog with carbon number equal to *i*.

**Fig. 1:**
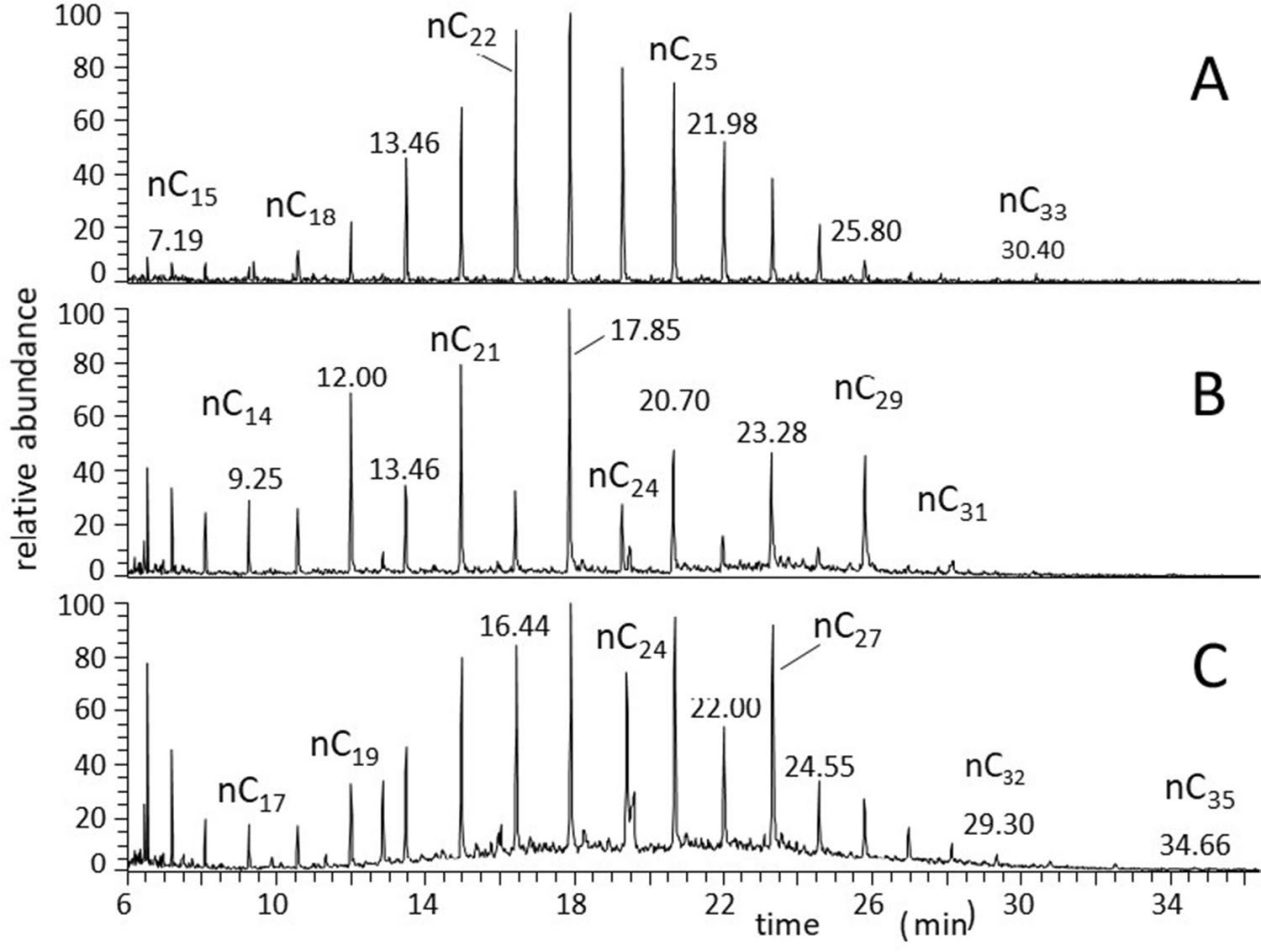
GC–MS profiles of the non-polar fraction (*m*/*z* = 85) of airborne particulates collected at four sites variously influenced by emission sources. **A** Road traffic site; **B** rural region; **C** urban location (city garden). Symbols: nC_*i*_ indicates the n-alkane with carbon number equal to *i*. [Personal communication. The samples were collected in the frame of a cooperative research project of our institute with INAIL-DIPIA, Rome, Italy, by applying the procedure described in Cecinato A, Marino F, Di Filippo P, Lepore L, Possanzini M (1999). Distribution of n-alkanes, polynuclear aromatic hydrocarbons and nitrated polynuclear aromatic hydrocarbons between the fine and coarse fractions of inhalable atmospheric particulates. *J Chromatog A* 846, 255–264, 10.1016/S0021-9673(99)00,129–6]

On the other hand, the n-alkane distribution typical of exhausts of fuels derived from petroleum is bell-shaped and mono-modal with the maximum centered between C_19_ and C_26_; in this case, CPI_25_ values range from 0.6 to 1.3 (Simoneit 1984; Perrone et al. 2014). According to that, CPI_25_ rates equal to ~ 1 were found during an in-field campaigns performed close to a highway in the Algiers metropolitan area (Fig. 1A), while CPI_25_ values were > 10 in a forest area belonging to Biskra province, Algeria (Fig. 1B). Anyway, usually a mix of the two distributions is observed, e.g., as it occurred in a city garden of Rome, Italy (Fig. 1C).

Marine biota behaves some differently. In fact, it retains the predominance of odd n-alkanes typical of biogenic emissions; however, the maximum shifts into the C_15_–C_21_ range. Thus, this percent distribution often characterizes the short/medium C-chain non-polar hydrocarbons collected at seaside locations (Romagnoli et al. 2016, Yu et al. 2019, Hernández-Guzmán et al. 2021, Gal et al. 2022); worth of note, this distribution is distinct from that associated to petrogenic sources, where the predominance of odd homologs is not observed.

Further indexes have been examined in order to put in evidence the impact of vegetation (Kumar et al. 2019). Among them, there are (i) the homolog (C_max_) corresponding to the maximum concentration within the n-alkane distribution; (ii) the cumulative percentage attributable to natural waxes (NW%) (Alves et al. 2001, 2011; Rabhi et al. 2018); and (iii) the average chain length of n-alkanes (ACL) (Leider et al. 2013). As for C_max_, motor vehicle exhausts exhibit the prevalence of short and medium chain hydrocarbons (< C_24_), while the leaf debris of high trees is characterized by the predominance of n-C_29_ or n-C_31_. The two distinct behaviors have pictured in Fig. 1A and B, where the maximums correspond to tricosane (C_23_) and nonacosane (C_29_), respectively.

The NW% value is provided by the formula (2):

2 $$\text{NW\%}={100}*\frac{{\sum }_{12}^{m}{\mathrm{nC}}_{2n+1}-\text{0.5*(}{\mathrm{nC}}_{2n+2}+{\mathrm{nC}}_{2n}\text{)}}{{\sum }_{12}^{m}{\mathrm{nC}}_{2n+1}}$$

where each term at the numerator is set equal to zero whereas the actual rate results < 0. For instance, NW% values ranging from ~ 10 up to > 70% have been calculated for airborne particulates collected in Athens metropolitan area and in Algeria (Andreou and Rapsomanikis 2009; Rabhi et al. 2018).

The average chain length of n-alkanes (ACL) is calculated through the formula (3):

3 $${\text{ACL}}=\frac{\sum n*\text{[}{\mathrm{nC}}_{n}\text{]}}{\sum [{\mathrm{nC}}_{n}]}$$

Carbon preference indexes analogous to CPI_25_ have been employed also, which consider longer n-alkane ranges (e.g., nC_11_ ÷ nC_36_) or the only light homolog segment (< nC_25_) (Alves et al. 2000, 2001; Aloulou et al. 2010). The use of CPIs computing light hydrocarbons is partly questionable due to volatility of compounds. However, these indexes allow investigating the possible impact of marine biota (algae, plankton) and microorganisms (bacteria, fungi), when molecular signature is extended to include isoprenoids and when nC_max_ corresponds to nC_15_/nC_17_ (Fisher et al. 1972; Ekpo et al. 2005; Andreou and Rapsomanikis 2009; Horikawa et al. 2010; Wang et al. 2010; Caumo et al. 2018). Table 1 provides a synthetic overview of what presented above. The action of marine organisms has been elucidated also as the possible source of squalene (2,6,10,15,19,23-hexamethyl-2,6,10,14,18,22-tetracosahexaene) and squalane in offshore and coastal airborne particulates, while abietane (13α-isopropylpodocarpane) and its homologs have been adopted to distinguish the emission of coniferous trees from that of other plants (Simoneit and Mazurek 1982; Fine et al. 2004).

**Table 1:** Indexes describing the n-alkane percent distributions, typical of various emission types. References: Simoneit (1984), Alves et al. (2001), Zrafi et al. (2008), Leider et al. (2013), Rabhi et al. (2018), Kumar et al. (2019)

Index/source	Petroleum	Algae	Microorganisms	High trees	Vegetation	Anthropogenic
C_max_	C_16_ ~ C_21_	C_17_, C_19_, C_21_, C_23_	C_16_, C_18_, C_20_	C_29_, C_31_, C_33_	C_25_, C_27_, C_29_	C_16_ ~ C_23_
CPI_25_	0.7–1.3		> 1	> 10	> 3	0.8–1.3
CPI_16_	0.8–1.2	> 1.0	> 1.0			
NW%	~ 0			> 75	> 75	~ 0–30

The bell-shaped and saw-teeth percent profiles are the two most common within medium/long-chain n-alkanes and are used commonly to identify the corresponding principal source. Nevertheless, sometimes a distinct fingerprint is observed in the semi-volatile range, where the even homologs are predominant. This pattern seems typical of sediments (Zrafi-Nouira et al. 2008, Sikes et al. 2009, Aloulou et al. 2010, Jafarabadi et al. 2018, Aghadadashi et al. 2021, Arshinova et al. 2021); nevertheless, it has been observed also in effluents from petroleum-contaminated zones and has been interpreted as a tracer of microorganisms including bacteria, fungi and spores (Stortini et al. 2009; Kuhn et al. 2010; Leider et al. 2013). Hence, the molecular signature over the whole nC_14_–nC_40_ range is more complex than as researchers currently believe and is not yet completely understood. For instance, it can exhibit high percentages of even homologs in the short/medium-chain range, and of odd homologs in the long-chain range.

Finally, the *R* ratio between total low molecular weight (LMW) and high molecular weight (HMW) n-alkanes seems to distinguish petrogenic emission (*R* >  > 1), terrestrial plants (*R* <  < 1), and marine biota (*R* ≈ 1).

Examples of in-field monitoring aimed at discriminating the sources of organic fraction of airborne particulates, sediments, and waters are Aghadadashi et al. (2021), Aloulou et al. (2010), Alves et al. (2001), Balducci et al. (2014), Bi et al. (2008), Gal et al. (2022), Kang et al. (2018), and Khedidji et al. (2017).

### Branched and cyclic hydrocarbons

The important presence of petroleum components (e.g., branched alkanes pristane and phytane) compared with nC_17_ and nC_18_, respectively, looks as a track of motor vehicle emission (Hamilton et al. 1984; Alexandrino et al. 2019; Alkhafaji 2021), or petrol spill from contaminated sediments, soils, and waters (Jeng 2006; Stortini et al. 2009; Wang et al. 2011; Shirneshan et al. 2016; Azimi-Yancheshmeh et al. 2017, Hernández-Guzmán et al. 2021). These ratios are adopted also to index the maturity of petroleum and bitumen affecting sediments (Omotoye et al. 2016; Gao et al. 2021).

Complex blends of high molecular weight aliphatic hydrocarbons with branched and/or cyclic structure (e.g., steranes and hopanes) are the bulk of petrol industry products, like fuels and solvents, and affect the exhausts (Xiao et al. 2019; Lu et al. 2021), soils and sediments (Arfaoui 2014). Their identification and quantification, combined with the ratio rates of burdens of subgroups, has revealed that composition depends on oil maturity or on progress of oxidative/biological attack of original blend (Lobodin et al. 2016; Volkman et al. 1997; Simoneit 1999). These compounds trace the environmental pollution associated with fossil fuels (Aboul-Kassim and Simoneit 1995, Fraser et al. 1997; Wang et al. 2006; Jedynska et al. 2014a,b; Iakovides et al. 2021a). Moreover, triterpanes and steranes seem suitable to characterize sedimentary organic matter and contaminated substrates (Arfaoui 2014; Xiao et al. 2019).

**Fig. 2:**
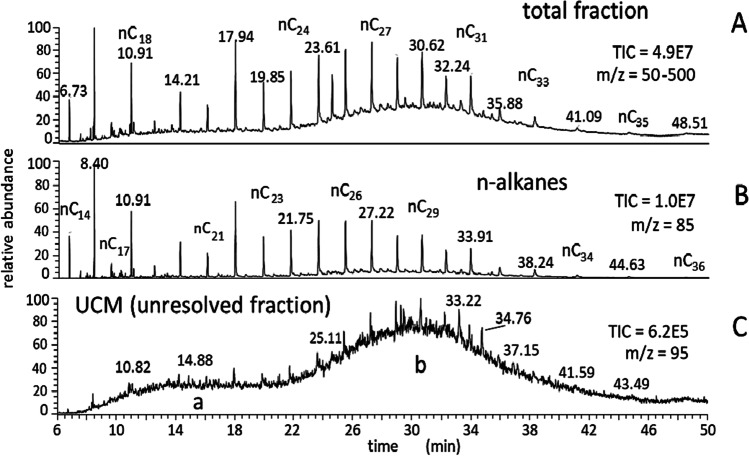
GC–MS profile of the non-polar fraction of diesel exhaust. **A** Total ion current signal; **B** Ion trace corresponding to *m*/*z* = 85 (labeling n-alkanes); **C** *m*/*z* = 95 ion trace (branched alkanes). Both *a* and *b* humps occur in the UCM. Symbols: nC_*i*_ indicates the n-alkane with carbon number equal to *i*. [Personal communication. The samples were collected in the frame of a cooperative research project of our institute with Istituto Motori CNR, Naples, Italy, by applying the procedure described in Ciccioli P, Cecinato A, Brancaleoni E, Draisci R, Liberti A (1989). Evaluation of nitrated polycyclic aromatic hydrocarbons in anthropogenic emission and air samples: a possible means of detecting reactions of carbonaceous particles in the atmosphere. *Aerosol Sci Technol* 10, 296–310, 10.1080/ 02786828908959266]

The composition profile of organic matters shows one or two humps of “unresolved mixture” accompanying the n-alkanes sequence (Zheng et al. 2002; Phuleria et al. 2006; White et al. 2013; Jeon et al. 2017). In the case of airborne particulate, a hump comprised of light hydrocarbons originates from gasoline and diesel oil residues. A second hump, including heavy components, is related to lubricating oils (see *a* and *b* humps in Fig. 2); the percent profile of vehicle emissions depends on the engine working conditions, and the relative importance of hump(s) raises at unregulated driving regimens, e.g., during cold starts (Zheng et al. 2002, Fang et al. 2020, Iakovides et al. 2021a, Tian et al. 2021).

Though unusual, high percentages of semi-volatile homologs (from C_20_ to C_26_) have been detected in airborne particulate coming overall from agricultural areas. This pattern has been found as typical of substrates contaminated with bee waxes (Guenther et al. 1995; Fine et al. 2004).

Among the sources of environmental non-polar hydrocarbons, both tobacco plant leaves and tobacco smoke fumes exhibit a peculiar percent profile with regard to monomethyl-branched alkanes. Indeed, long-chain odd iso-alkanes and even anteiso-alkanes are much abundant compared to normal-alkanes than in other emissions; in particular, anteiso-C_30_/C_32_ are more than normal-C_30_/C_32_, respectively (Kavouras et al. 1998). Besides, the normal hentriacontane (nC_31_) is predominant when compared to nC_29_ and nC_33_ homologs [nC_31_/average(n-C_29_, nC_33_) > 1.5] (Cecinato et al. 2022). This twin molecular signature was observed in tobacco smoke chambers and in interiors heavily contaminated by smoke and allowed to derive a semi-quantitative index (%ETS) suitable for estimating the percentage contribution of tobacco smoke in airborne particulates and depositions (Cecinato et al. 2022). The %ETS is calculated by applying the formula:

$$\%\mathrm{ETS}=\mathrm{TSI}\ast\%_0\left(\Sigma_{\mathrm{As}}\right)/25.8$$

where:

$$\mathrm{TSI}={~}^{1}\!\left/ \!{~}_{3}\right.*(\frac{A}{1.36}+\frac{B}{9.85}+\frac{C}{1.29})*100$$

and:

aC_*j*_, iC_*j*_, and nC_*j*_ are the anteiso-, iso-, and normal-C_*j*_ alkane, respectively.

*A =*: 1/6 (iC_29_/nC_29_ + aC_30_/nC_30_ + iC_31_/nC_31_ + aC_32_/nC_32_ + iC_33_/nC_33_ + aC_34_/nC_34_),
*B =*: 1/6 (iC_29_/aC_29_ + aC_30_/iC_30_ + iC_31_/aC_31_ + aC_32_/iC_32_ + iC_33_/aC_33_ + aC_34_/iC_34_),
*C =*: 1/6 (aC_30_/nC_30_ + aC_32_/nC_32_ + aC_34_/nC_34_ + aC_29_/nC_29_ + aC_31_/nC_31_ + aC_33_/nC_33_),
‰(Σ_As_): per thousand content of total alkanes in particulate matter.

(Rem.: For the meaning of 2.58, 1.36, 9.85 and 1.29 at denominators see Cecinato et al. 2022).

Total uncertainty of %ETS is provided by:

$${\mathrm S}_{\%\mathrm E\mathrm T\mathrm S}=100\ast\mathrm{std}.\mathrm{dev}.\left(\mathrm A,\mathrm B,\mathrm C\right)\ast\permille\left(\Sigma_{\mathrm{As}}\right)/25.8$$

Worth of note, this approach does not require searching for minor markers like nicotelline and nitrosamines, nor for nicotine and cotinine, which are ease to decompose and volatilize (see “Fatty acids and alcohols, polar compounds” section).

### Alkenes

**Fig. 3:**
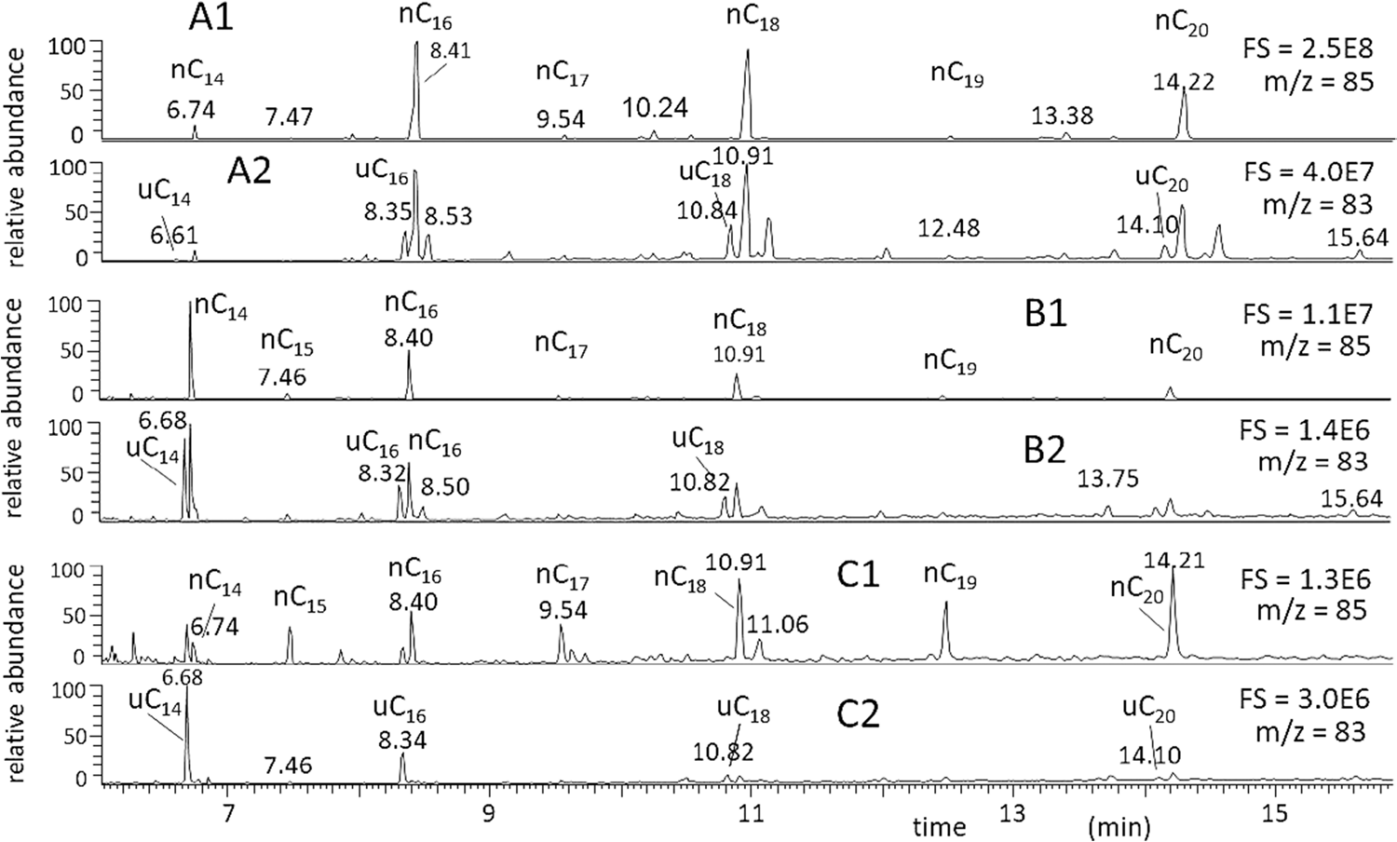
GC–MS chromatograms of airborne particulate extracts. **A1** El Bey, Tunisia, *m*/*z* = 85 (n-alkanes); **A2** El Bey, Tunisia, *m*/*z* = 83 (n-alkenes); **B1** Tipaza, Algeria, *m*/*z* = 85 (n-alkanes); **B2** Tipaza, Algeria, *m*/*z* = 83 (n-alkenes); **C1** Reggio Calabria, Italy, *m*/*z* = 85 (n-alkanes); **C2** Reggio Calabria, Italy, *m*/*z* = 83 (n-alkenes). [Personal communication. The samples were collected in the frame of a cooperative research project of our institute with Kasdi Merbah University of Ouargla, Dept. Mathematics and Sciences of Matter, Touggourt, Algeria, by applying the procedure described in Romagnoli P, Balducci C, Perilli M, Perreca E, Cecinato A (2016). Particulate PAHs and n-alkanes in the air over Southern and Eastern Mediterranean Sea. Chemosphere 159, 516-525. https://doi.org/10.1016/j.chemosphere.2016.06.024]

Though scarce attention is paid to unsaturated hydrocarbons occurring in emissions, three distinct molecular signatures of normal alkenes can be distinguished within the light range of non-polar fraction of POM (i.e., mono-unsaturated hydrocarbons with 12 up to 20 carbon atoms in the molecule) (Ekpo et al. 2005). They are (i) the predominant occurrence of n-alkanes, with negligible amounts of n-alkenes; (ii) the prevalence of n-alkenes, displaying high dodecene/dodecane and tetradecene/tetradecane ratios, and low octadecene/octadecane and eicosene/eicosane ratios; and (iii) a merged distribution. The three fingerprints are pictured in Fig. 3. Sample A (deposition dust collected indoors at El Bey, Tunisia) was comprised of much more n-alkanes than alkenes (Fig. 3A1/A2); dusts from Tipaza, Algeria (Fig. 3B1/B2), comprised both alkanes and alkenes; finally, n-alkenes prevailed on n-alkanes in depositions collected in Reggio Calabria, Italy (Fig. 3C1/C2).

It is known that unsaturated hydrocarbons occur as minor components in vegetation emissions; nevertheless, to our knowledge no exhaustive explanation of the three patterns abovementioned has been found until now, nor specific investigations have been undertaken concerning the alkene fingerprints in aerosols and dusts. The fact that in the correspondence of alkene predominance vs. alkanes the even homologs are much more than the odd ones seems to suggest that all compounds enjoy of the same biogenic source; otherwise, alkenes would originate from the twin decarboxylation of unsaturated even α,ω-dicarboxylic acids. Anyway, further investigations are necessary to confirm this hypothesis and search for alternative solutions. For sake of completeness, it is worth to note that the occurrence of ≥ C_20_ alkenes and polyalkenes in estuarine and coastal sediments, even exceeding the corresponding n-alkanes, has been associated with algae and phytoplankton (Requejo and Quinn 1983; Yongdong et al. 2015).

### Polycyclic aromatic hydrocarbons

Though accounting for small fractions of organic matter, PAHs are of big concern, because of their strong toxicity in terms of carcinogenic and mutagenic power as well as of their ability to promote heart morbidity and premature deaths (Collins et al. 1998, European Parliament and Council 2005, IARC 2012). Organic particulates exhibit a plurality of PAH signatures, and many attempts have been made to associate the PAH chemical imprinting with the nature of emission; this was important not only in the perspective of assessing environmental toxicity, but also with regard to forensics sciences and remediation policies (Andersson and Achten 2015; Stout et al. 2015). It is worth to remark that the original percentages of PAHs in emissions do not correspond exactly to those found in PM and dust (Kavouras et al. 1999; Kim et al. 2009; Tobiszewski and Namiesnik 2012; Keyte et al. 2013; Stogiannidis and Laane 2015; Emsbo-Mattingly and Litman 2016). Indeed, most PAHs are released at hot conditions by organic matter that burns (e.g., during forest fires and fuel combustion), and originally exist as vapors; thereafter, PAHs condense onto solid substrates according to vapor pressure of compounds, to temperature and substrate features; besides, fuel spill and evaporation occur from petroleum reservoirs and bitumen/asphalt (Alves et al. 2011). As long as adsorbed, PAHs share with particles the ways of dispersion in air, including long-range transport, and finally settle onto surfaces together with coarse dust (Simoneit 2002; Medeiros and Simoneit 2008; Ravindra et al. 2008; Lammel et al. 2010; Iakovides et al. 2021b). The gas/particle equilibrium developing at the particle surface is dynamic and is influenced by reactivity of congeners, though the principal PAHs (e.g., the sixteen included in the list of priority pollutants (USEPA 1993)) are classified as persistent toxicants (USEPA 1993; MacKay and Callcott 1998; Lodovici et al. 2003; Paolini et al. 2015; Cao et al. 2019), and reactivity changes when the compounds are adsorbed on carbonaceous substrates or silica (Keyte et al. 2013, and references herein). In conclusion, as underlined by several authors, both the collection parameters (e.g., time of sampling, presence of ozone/oxidant traps, fiber membrane material, etc.) and chemical analysis procedure modulate the resulting concentrations of individual PAHs in the particulates (Tobiszewski and Namiesnik 2012; Balducci et al. 2017).

Only a handful of PAHs, individually or as subgroups, have been associated to specific sources. Among them, worth of mention are retene (1-methyl,7-isopropylphenanthrene), methylphenanthrenes, dimethyl/ethyl-phenanthrenes, and benzo[ghi]fluoranthene (Tong and Karasek 1984; Benner et al. 1995; Shen et al. 2012). In fact, retene is typical of wood and is a common tracer of forest fires; dimethyl/ethyl-phenanthrene isomers occur as mixtures that display distinct profiles in the case of vegetation and fossil fuel combustion; and benzo[ghi]fluoranthene is an important component of motor vehicle exhausts. Hence, the dimethyl/ethylphenanthrene molecular fingerprint in atmospheric particulate results depending on the daily and seasonal modulation of sources (Paolini et al. 2015). On the other hand, only very fresh emissions hold benzo[b]anthracene (naphthacene) and anthanthrene, both prone to fast decomposition (Wise et al. 1988; Dominguez et al. 2003; Kim et al. 2009). In fact, the occurrence of the two compounds was documented in exhausts but was not regularly in the atmosphere. The methylphenanthrene mixture displays distinct isomer distributions in accordance with the kind of the emission source. Moreover, analogously to all other alkyl-PAH/parent PAH ratios, the rate of methylphenanthrenes/phenanthrene ratio depends on the predominance of kerosene spill or oil combustion exhausts, and on burning temperature and time. This ratio is also an index of thermal maturity of oil samples (Omotoye et al. 2016).

Normally, numerous PAHs affect particulates, and researchers prefer analyzing concentration ratios between pairs of individual substances to draw information about the nature of emissions (Brandli et al. 2007; Ravindra et al. 2008; Katsoyiannis et al. 2011; Katsoyiannis and Breivik 2014; Famiyeh et al. 2021). Concentration ratios that overall look as diagnostic for this purpose are fluoranthene vs. pyrene (FA/PY), benz[a]anthracene vs. chrysene (BaA/CH), indeno[1.2.3-cd]pyrene vs. benzo[ghi]perylene (IP/BPE), and benzo[a]pyrene vs. benzo[ghi]perylene (BaP/BPE). Other ratios, e.g., phenanthrene vs. anthracene (PHE/AN), total methylphenanthrene vs. phenanthrene (ΣMPHE/PHE), and unsubstituted PAHs vs. total PAHs including alkyl-substituted congeners (parent-PAHs/ΣPAHs), are investigated less frequently. Table 2 provides a list of PAH diagnostic ratios (DRs) and the respective values calculated for several categories of emissions, as described by scientific literature. As shown in Table 2, the DR values associated to various emissions are affected by some variability; therefore, to investigate the sources of particulate PAHs the authors adopted, in field experiments, both short DR ranges and more DRs simultaneously (usually three/four pairs) instead of one only DR and one precise DR rate (Famiyeh et al. 2021). The benzo[a]pyrene/benzo[e]pyrene ratio (BaP/BeP) merits a special comment. Indeed, BeP was neglected for long time, because it is much less carcinogenic than other PAHs; this is the reason why BeP does not appear among the sixteen priority PAHs. On the other hand, BeP occurs in emissions at similar extents as benzofluoranthenes and it is more persistent than benzo[a]pyrene. Thus, the concentration ratio between BaP and BeP is usually ≈1.0 in fresh exhausts but tends to drop slowly to < 0.1, overall in the presence of oxidants. For instance, in two PAH monitoring campaigns carried out in Milan, Italy, during 1991, at a site exposed predominantly to vehicle traffic (Cecinato 1997), the BaP/BeP ratio reached 0.9 in the winter and was as low as 0.1 in the summer. The information obtained through molecular signatures of PAHs alone seems insufficient to quantify the contribution of each emission source to the whole of environmental particulates; however, it helps in recognizing the principal causes of pollution and can be improved through associating other markers like oxy-PAHs, sugars, and alkanes (Zheng et al. 2002; Tian et al. 2021; Shin et al. 2022). Besides, this approach allows highlighting the role of oxidation processes with regard to toxicity, whenever the final products (e.g., PAH quinones and lactones) are more harmful than their parent compounds (Durant et al. 1999).

**Table 2:** Rates of the principal PAH concentration ratios currently used as diagnostic tools to draw insights about the nature of source. References: Kavouras et al. (2001), Ravindra et al. (2008), Tobiszewski and Namiesnik (2012), Cecinato et al. (2014), Famiyeh et al. (2021)

Source	Type	FA/PY	BaA/CH	IP/BPE	BaP/BPE	BaP/BeP
Vehicles	Mixed	0.60			0.55	
	Gasoline	0.54	0.8–1.3	0.20–0.35	0.35	0.95
	Diesel	0.8–1.1	0.38	0.65–1.1	0.8–1.1	0.50
Domestic heating	Coal		0.65	0.9–1.3	1.57	
	Wood, pine	0.78	0.64	1.1–1.6	1.94	2.1
	Wood, oak	0.75	0.70	1.2–1.6	1.77	1.77
	Synthesis fuel	1.19	0.78	1.1	1.91	
	Heavy oil	0.83	1.01	1.6	0.81	0.52
Steel plant	Coke		0.65	0.9–1.3	1.57	
	Power (coke)	0.66	0.56	2.0	0.88	2.57
Tobacco smoke	Particulate	0.96	1.3	0.18	0.23	0.38
Waste fumes	Landfill	1.3	0.84	0.76	0.70	0.55
	Incinerator	≈17	0.71	0.92	~ 0.12	0.01

### Nitrated polycyclic aromatic hydrocarbons (NPAHs)

NPAHs begun of big concern when many chemicals belonging to this group were identified in emissions (Hoekman 1992; Zielinska et al. 2004; Liu et al. 2010). In particular, the huge increment of diesel engine vehicles during 1970s and 1980s contributed to the occurrence of NPAHs in the air of cities worldwide. Besides, diesel engines were ascertained as main sources of NPAHs (Bamford and Baker 2003; Bandowe et al. 2014; Bandowe and Meusel 2017); on the other hand, many NPAHs were recognized as direct mutagens and cancer promoters (Gbeddy et al. 2020). The occurrence of NPAHs in the air declined with the updating of normative dealing with vehicle emissions and the consequent renewal of vehicle fleets; hence, NPAHs lost concern and their measurements in the environment dropped since 1990s. Instead, NPAH investigations have started again in recent years, due to their toxic properties (Degrendele et al. 2021) and to occurrence in gasoline-fueled cars (Zhao et al. 2020). Usually, attention is paid to a list of NPAHs affecting airborne particulate; they are nitrated derivatives of naphthalene, fluorene, anthracene, fluoranthene, pyrene, benz[a]anthracene, and chrysene. Nevertheless, NO_2_-position isomeric PAHs associated to airborne particulates are not the principal ones coming out from emissions. In particular, 2-nitrofluoranthene and 2-nitropyrene are commonly absent in exhausts and exist as products of in situ reactions developing in the atmosphere; the two compounds are sometimes the most abundant NPAHs affecting particulates (Bamford and Baker 2003; Bandowe and Meusel 2017).

The molecular signature of nitrated fluoranthenes and pyrenes has been used to parameterize the relative importance of direct emission and action of oxidants. Taking in account the nitration rate of precursors reacting with OH radicals and NO_2_, it was suggested that photochemical reactivity is more important than vs. direct emission when the 2-NFA/1-NPY ratio exceeds 5.0 (Pitts et al. 1985). Instead, the formation of 4-NPY can occur only in the presence of NO_3_ radical or N_2_O_5_; thus, this isomer is a tracer of processes started by reaction of O_3_ with NO_2_ and developing after sunset. Analogously, distinct nitro-isomers are formed by homogeneous and heterogeneous reactions of other parent PAHs with NO_2_, OH + NO_2_, and NO_3_/N_2_O_5_ (Jariyasopit et al. 2014, and references herein).

### Fatty acids and alcohols, polar compounds

Medium- and long-chain acids exist overall thanks to living organisms releasing them (Goutx and Saliot 1980; Kawamura and Gagosian 1987; Lindbeck and Puxbaum 1999; Oliveira et al. 2007; Bi et al. 2008; Sangiorgi et al. 2013; Balducci et al. 2014). The percent distribution pattern of fatty acids reveals the clear prevalence of even carbon atom homologs, and the rates of carbon preference indexes (ACPIs), formulated similarly to those of n-alkanes, usually exceed 10 (Alves et al. 2001). Other sources show analogous profiles; e.g., vehicle exhausts hold A_12_–A_22_ acids (i.e., linear chain homologs with 12 ÷ 22 carbon atoms), with the maximums corresponding to A_16_ and A_18_. Biogenic emissions show also typical percentages of medium- and long-chain fatty acids. Usually, apart from palmitic (A_16_) and stearic (A_18_) acids, the profiles display a secondary maximum within the ranges A_20_ ÷ A_24_, or > A_25_; light homologs have been associated with microbiota, small plants, and softwood trees, while heavy acids characterize high plants and hardwood trees (Gelpi et al. 1970). Worth of note, the ACPI rates are lower in the case of microorganisms, due to important percentages of odd-carbon acids from A_15_ to A_21_.

Unsaturated and dicarboxylic acids merit a special mention. Unsaturated acids (UA_*n*_), e.g., palmitoleic (UA_16_), oleic (UA_18_), and linoleic (twin unsaturated A_18_), are indicative of emission from crops and in interiors of cooking (Schauer et al. 2002; Yu et al. 2021). Dicarboxylic fatty acids (DA_*n*_) exist as minor components released by vegetation, whose emissions show the usual even-to-odd carbon prevalence; however, particulate matters are rich of DA_2_–DA_6_ homologs, emitted by anthropogenic sources, which influence the molecular signature of the short C-chain range. Besides, air parcels affected by oxidants show the occurrence of azelaic acid (DA_9_), which is a by-product of oleic acid decomposition (Balducci et al. 2014; Kawamura and Bikkina 2016; Ren et al. 2020).

Linear alcohols, 2-ketones, aldehydes, fatty acid methyl esters, and nitriles were not extensively investigated as tracers of suspended particulate sources, though all of them have been linked to emission from vegetation (Simoneit and Mazurek 1982; Simoneit 2002) and have been found in pyrolysis by-products of sewage sludge treated with aerobic and anaerobic digestion (Dominguez et al. 2003). Linear alcohols exhibit a behavior parallel to that of fatty acids. They display the predominance of even carbon homologs and are among the principal components of organic aerosols in rural regions (Simoneit and Mazurek 1982). Many sterols also (including campesterol, sitosterol, stigmasterol, and amyrins) have been associated to vegetation as components of epicuticular waxes (Guo et al. 2019; Kumar et al. 2019; Gal et al. 2022). The most important exception is cholesterol, which has been recognized as a tracer of meat cooking (Cass 1998; Carreira et al. 2009). Other acid esters (including biopolymers, benzoates, terephthalates, myristates, and glycols) are employed nowadays as surrogates of old components of plastics (Sanchez-Pinero et al. 2021; Evtyugina et al. 2021), because these latter have been classified as emerging contaminants (Cavanagh et al. 2018, Udayakumar et al. 2021). Thus, the occurrence of new esters in soot and dust would be indicative of contamination by new plasticizers and, in interiors, of house cleaning, painting and building commodities as well as of cosmetics and other personal care products. No extensive investigations have undertaken in the environment regarding this topic; however, they should be gain importance in the future, due to the general tendency to replace alkyl phthalates and polyvinyl chloride with eco-friend plasticizers.

Several polar organics, including nitrosamines and nicotelline, have been suggested in the last decade as tracers of mainstream, sidestream, and third-hand tobacco smoke (Apelberg et al. 2013; Blanchard et al. 2014) in addition to, or as substitute for, nicotine, cotinine, fine particulate, and CO (Hecht 1999; Hammond et al. 1987; Daisey 1999). In particular, nicotelline has recognized as fine to perform quantitative assessments of the tobacco smoke contribution to pollution of indoor and outdoor environment, thanks to its low volatility and enough persistence in the air (Aquilina et al. 2021).

### Organic halides

Polychlorobiphenyls (PCBs) and polychlorinated dioxins/furans (PCDD/Fs) are probably the most investigated groups of halides affecting the environment (Barbas et al. 2018). PCBs were important industrial products during the twentieth century, since they found a number of applications as mixtures as solvent, in power transformers and heat exchangers, in substrates for pesticides and inks. By contrast, PCDDs and PCDFs exist solely as unwanted by-products of other industrial processes (e.g., paint manufacturing, foundries and steel mill, waste incineration). Despite PCBs and PCDD/Fs have been banned since long time, both groups continue to affect the environment today again (Ngo et al. 2020). Industrial syntheses of PCBs lead to blends characterized by various average chlorine percentages, which sometimes could aid in highlighting the impact of sources suspected as causing environmental pollution. A variety of fingerprints tags the emission sources; for instance, distinct PCDD/Fs patterns have observed for vehicle exhausts, sewage sludge, and steel mill fumes (Mininni et al. 2004; Liu et al. 2015). Besides that, the molecular fingerprints of PCBs and PCDD/Fs change with time owing to ability of congeners to persist to degradation as well as to dissolve in waters and lipids (Di Guardo et al. 2017; Ngo et al. 2018, 2020). Finally, looking to bioavailability of organic halides, we must take in account that these substances are semi-volatile. For instance, 2.3.7.8-tetrachlorodibenzo-p-dioxin exists overall as vapor in the environment, while most dioxin-like congeners occur as adsorbed on particulates (Barbas et al. 2018). Due to key role of dioxin-like compounds regarding to toxicity, the contemporary collection of gaseous and condensed phases of emissions and atmosphere is mandatory to draw information about sources and air quality. Nevertheless, the actual risk for humans depends on the aggregation state of toxicants, because vapors are in part breathed out, while ultra-fine and fine particles are easily retained in lungs with their harmful load.

Other halides have recently gained concern as tracers of water, air, and soil pollution depending on waste spill and contaminated food. In particular, polybromodiphenyl ethers (PBDEs) and phosphoric acid organic esters are present in flame retardant formulas (Lee et al. 2020; Percy et al. 2020), while perfluoroalkyl acids (PFAs) and other perfluorinated chemicals enjoy of many industrial and home care applications as surfactants (Hubbard et al. 2012).

### Polysaccharides

The occurrence of numerous organic substances in the environment is associated with biomass burning in general, and specifically with that of specific tree species (Oros and Simoneit 2001a, b, Oros et al. 2002). Many chemicals are carbohydrate molecules (e.g., glucose, xylose, and sucrose) and the respective dehydration-polymerization by-products of them (levoglucosan, galactosan, mannosan, inositols) (Simoneit et al. 2004; Jia and Fraser 2011; Pereira et al. 2017; Bikkina et al. 2019; Lv et al. 2021) and of lignin (e.g., methoxyphenols) (Hawthorne et al. 1988; Hays et al. 2005). Levoglucosan is usually the predominant anhydrosugar; however, other minor polysaccharides allow distinguishing hardwood from softwood burning, thanks to their concentration ratios vs. levoglucosan; in fact, levoglucosan/mannosan ratios ranging from 3 to 10 are typical combustion of softwood, while ratios ranging 15 ÷ 25 of hardwood, and > 40 of crop burning (Kang et al. 2018; Mu et al. 2021). Levoglucosan in particular, typical tracer of wood burning, allowed to demonstrate that even the downtowns of big cities undergo the impact of this kind of emission, due to the generalized use of wood for heating and cooking in the countryside (Fine et al. 2004). Indeed, wide uncertainty remains about the emission rate of these chemicals; nevertheless, according to levoglucosan levels in air, manmade biomass burning looks as the principal source of pollution outside of cities and heavy industry districts (Pomata et al. 2014; Perrino et al. 2019; Ren et al. 2020).

## Living organisms as unexpected and unconsidered sources of organic contaminants

All living organisms, including humans, are not only the target, but also the source of a number of contaminants (Settimo et al. 2020b). Microorganisms are exploited to remove organic toxicants through aerobic and anaerobic digestion (Habib et al. 2022; Priya et al. 2022). On the other hand, fungi, spores, bacteria, insects, and indoor plants inhabit our environment, leave everywhere traces of their presence, and often move people to use repellants, pesticides, and disinfectants; meanwhile, pets are the source of allergies, irritations, and breathing problems (Diaz 2016; Zhai et al. 2018; Settimo et al. 2020b; Cui et al. 2022). This phenomenon is much more important in building interiors, where humans contribute in a twin way, i.e., (i) indirectly, through actions related to use of home and personal care products (deodorants, cleaning sprays, soaps, fragrances, plastics), to cooking (foods), wearing (fibers, dyes), and heating/air conditioning (fuels, freezing liquids); and (ii) directly, through emitting vapors, droplets, and particles (e.g., through breath, sweat, hair loss, skin abrasion) (Nazzaro-Porro et al. 1979; Bortz et al. 1989; Camera et al. 2010; Knox and O’Boyle 2021). Chemical composition of man’s skin and hair lipids, sweat, and breath is known since long time; however, the concern about it seems restricted to industry of cosmetics and related production, while at our knowledge no investigations are undertaken concerning their occurrence in our life places. Hence, the search for specific molecular fingerprints that allow indexing their impact on the chemistry of the environments is still at the start point.

## Conclusions

The sources of pollution (both direct emissions and atmospheric reactions leading to presence of toxicants in the environment) model the molecular fingerprint of organic contaminants associated to airborne particulates and deposition dusts. This chemical signature is comprised of individual markers and, more often, of distribution patterns within groups of homologs. Both types of signature provide preliminary but useful information about nature of emissions and with regard to their health impact on environment. Though studied since long time, the molecular fingerprints of emissions are not completely elucidated and further investigations seem necessary, due to recent detection of new distribution models of particulate matter components that mess up consolidated behaviors (e.g., alkanes), as well as to the novel concern for emission sources neglected until now (microorganisms). The progress of knowledge about the molecular fingerprints of sources will aid investigators to apply more sophisticated approaches (e.g., providing explanation of the crude results of principal component analysis or source factorization modeling) and assess the relative importance of emissions. Besides, it will favor optimizing the strategies aimed at controlling air pollution and mitigating the impact of toxicants on humans and environment. In that perspective, new studies are advisable to do, aimed at characterizing chemicals released by humans, pets, and living microorganisms, which often prejudice the healthiness of the life places.

## Data Availability

The datasets used and/or analyzed during the current study are available from the corresponding author on reasonable request. Anyway, those not directly produced by personal investigations of authors are available in the bibliography cited in the paper.
